# Metabolic Profile of Alzheimer’s Disease: Is 10-Hydroxy-2-decenoic Acid a Pertinent Metabolic Adjuster?

**DOI:** 10.3390/metabo13080954

**Published:** 2023-08-18

**Authors:** Yuan Gong, Hongjie Luo, Zeju Li, Yijun Feng, Zhen Liu, Jie Chang

**Affiliations:** Department of Occupational and Environmental Health, School of Public Health, Soochow University, 199 Ren’ai Road, Suzhou 215123, China; bravogary@yandex.com (Y.G.);

**Keywords:** Alzheimer’s disease, diabetes mellitus, obesity, 10-HDA, molecular docking

## Abstract

Alzheimer’s disease (AD) represents a significant public health concern in modern society. Metabolic syndrome (MetS), which includes diabetes mellitus (DM) and obesity, represents a modifiable risk factor for AD. MetS and AD are interconnected through various mechanisms, such as mitochondrial dysfunction, oxidative stress, insulin resistance (IR), vascular impairment, inflammation, and endoplasmic reticulum (ER) stress. Therefore, it is necessary to seek a multi-targeted and safer approach to intervention. Thus, 10-hydroxy-2-decenoic acid (10-HDA), a unique hydroxy fatty acid in royal jelly, has shown promising anti-neuroinflammatory, blood–brain barrier (BBB)-preserving, and neurogenesis-promoting properties. In this paper, we provide a summary of the relationship between MetS and AD, together with an introduction to 10-HDA as a potential intervention nutrient. In addition, molecular docking is performed to explore the metabolic tuning properties of 10-HDA with associated macromolecules such as GLP-1R, PPARs, GSK-3, and TREM2. In conclusion, there is a close relationship between AD and MetS, and 10-HDA shows potential as a beneficial nutritional intervention for both AD and MetS.

## 1. Introduction

DM is characterized by chronic hyperglycemia that results from disturbed insulin secretion or insulin dysfunction, or both [1]. Reportedly, in 2018, a total of 440 million people worldwide were living with DM [2], and China and India were the countries with the highest rates [3]. It is estimated that this number will see a worldwide increase of 202 million by 2040 [3]. According to a nationwide population-based study, individuals recently diagnosed with DM have an increased risk of developing AD after an 11-year follow-up period [4]. Furthermore, an epidemiological study suggests that type 2 diabetes mellitus (T2DM) is a critical independent risk factor for neuropsychological symptoms, inducing anxiety, depression, and appetite disturbance in early AD [5]. The strong association between AD and DM has led many researchers to refer to AD as “type 3 DM” [6].

Moreover, obesity is a risk factor of T2DM. Obesity is a global public health problem, the incidence of which has tripled since 1975 [7]. It is a health condition defined as excess body fat that endangers one’s health. In clinical practice, it is evaluated by the body mass index (BMI), calculated as weight (kg)/height^2^(m^2^), and is diagnosed at a BMI ≥ 30 kg/m^2^ according to the WHO [8].

AD is the primary cause of dementia, which is characterized by progressive memory loss and cognitive dysfunction. According to global data, it is estimated that 50 million people were living with dementia in 2018, and this is suggested to triple by 2050 [9]. According to current knowledge, AD is a neuron-jeopardizing disease, which brings about a gradual loss of capacity to carry out everyday functions such as walking and gripping. Furthermore, it can lead to coma and eventually, death [10]. All the above pose a considerable challenge to society. In addition, the risk of developing AD increases from 50% to 75% in people with DM [11], especially T2DM [12]. Moreover, the likelihood of a person having AD increases when their BMI is higher than average, especially if they have both obesity and poorly controlled DM [13,14]. Notably, T2DM and obesity originate from an unhealthy diet and lifestyle, which means that intervention through personal, clinical, and public health means is practical.

Through several interacting mechanisms, obesity and T2DM progressively cause tissue damage, particularly in the hippocampus, ultimately resulting in a decline in overall health [15,16,17]. Among the mechanisms linking MetS to AD, low-grade chronic inflammation plays a critical role in the pathogenesis of AD. Importantly, low-grade inflammation disarms the BBB, making brain cells vulnerable to external stimuli [18,19].

Royal jelly (RJ), a white or yellowish gelatinous substance, is produced by the hypopharynx and mandibular salivary glands of honeybees [20]. It has a long history of use in traditional Chinese medicine due to its various benefits, including its antioxidative, antimicrobial, anti-inflammatory, immunomodulatory, neurotrophic, and MetS-preventing activities [21,22]. Regarded as a marker of RJ quality, 10-HDA is a distinctive, unsaturated fatty acid that is present in RJ [23], and it may be involved in the biological activities of RJ, such as antitumor and antimicrobial activities [24,25]. Moreover, recent research has found that 10-HDA exhibits various advantageous characteristics, such as anti-inflammatory, immunomodulatory, antimicrobial, antitumor, antioxidative, and vessel-preserving properties [26]. These properties make 10-HDA a potentially valuable nutrient for addressing AD, particularly AD associated with MetS.

This article aims to elucidate the current understanding of the relationship between AD, DM, and obesity, and subsequently introduces 10-HDA as a potential intervention nutrient, supported by bioinformatic analysis.

## 2. Metabolic Syndrome and Alzheimer’s Disease

**Figure 1:**
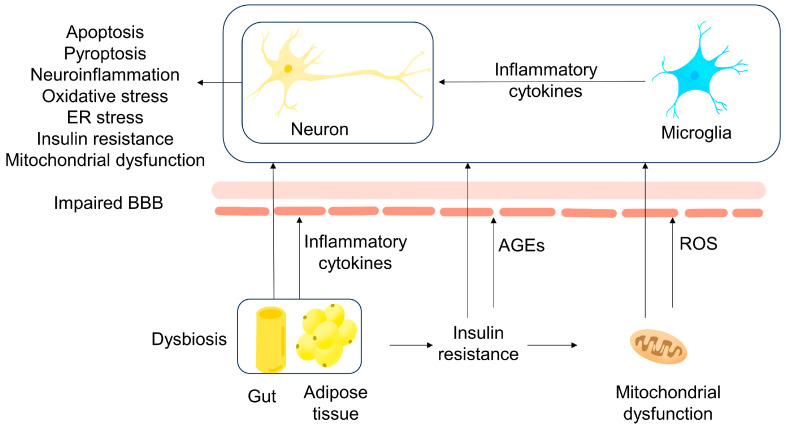
A summary of the pathogenesis of MetS-related AD. First, under low-grade inflammation in adipose tissue caused by MetS, IR is formed in the adipose tissue, liver, and muscles due to impaired tyrosine phosphorylation. Afterwards, IR induces hyperglycemia, which causes the formation of more AGEs. IR also leads to energy metabolism dysfunction in mitochondria, which causes more ROS production. Released inflammatory cytokines, AGEs, and ROS damage the BBB; then, they enter the brain matter via the impaired BBB. After entering, they directly hamper neurons or activate microglia cells to damage neurons.

The WHO defines MetS as a state characterized by IR, abdominal obesity, hypertension, and hyperlipidemia. The brains of individuals with AD-related dementia also seem to develop IR, according to research published in recent years [27]. This means that MetS might not only be a risk factor of AD, but it might also play a role in the pathogenesis process [28]. Considering the above, interventions targeting MetS may be beneficial in preventing and treating AD [29]. A summary is shown in Figure 1.

### 2.1. Type II Diabetes Mellitus

DM is recognized as the primary form of MetS. A study conducted as part of the AD Neuroimaging Initiative suggests that the level of amyloid β1-42 increases in the cerebrospinal fluid of T2DM patients compared to nondiabetic patients, while the opposite pattern is observed in the cerebral cortex [30]. Additionally, another observational study shows that the level of total tau is raised in APOE E4^+^ AD patients with DM [31]. According to a data-driven modeling study, genes such as ABCG1, COMT, MMP9, and SOD2 may play roles in T2DM and AD, and are related to biological process such as IR, oxidative stress, apoptosis, and cognition [32]. Using bioinformatics analysis, Huang et al. discovered that T2DM and AD are closely related through microvascular complications [33]. In a study searching for biomarkers such as insulin, HbA1c, and lipid profile disturbance, it was suggested that CRP and D-dimer play a role in the bilateral pathogenesis between AD and T2DM [34]. These findings further emphasize the interconnectedness between AD and DM.

T2DM is distinguished by a gradual beta cell failure and IR, and IR is the primary mechanism that relates DM to AD. Moreover, BBB injury, inflammation, oxidative stress, mitochondrial dysfunction, glucose metabolism dysfunction, AGEs, and vascular dysfunction also play a role in the interplay between DM and AD [35].

#### 2.1.1. Insulin Resistance

Insulin plays a crucial role as a metabolic regulator in the brain. Firstly, insulin secreted by pancreatic beta cells can find its way across the BBB or the blood–cerebrospinal barrier through insulin transporters. Additionally, certain regions of the brain are capable of producing and releasing their own insulin, although majority of insulin in the brain is believed to be derived from the pancreas [36]. Secondly, the brain itself is an insulin-sensitive organ. The brain expresses insulin receptors in various regions, such as the olfactory bulb, cerebral cortex, hypothalamus, cerebellum, and hippocampus [37]. Thirdly, after insulin binds to the insulin receptors, they phosphorylate the insulin receptor substrate. Then, the insulin receptor substrate activates PI3K and downstream MAPK pathways. The activated PI3K upregulates Akt, and then Akt downregulates GSK-3β, mTOR, and forkhead box O (FOXO). Eventually, PI3K signaling regulates protein synthesis, inflammation, mitochondrial dysfunction, autophagy, cell survival, and proliferation, while the MAPK pathway regulates proliferation, inflammation, and apoptosis [38]. Furthermore, the activation of JNK pathways (a subtype of the MAPK pathway) can inhibit PI3K signaling and lead to IR. Increased intracellular Ca^2+^ has been proposed as a possible mechanism that affects the localization and protein–protein interactions of key signaling mediators, explaining both JNK activation and the inhibition of PI3K signaling [39].

IR is a condition in which insulin-targeted tissues such as the liver, adipose tissue, and skeletal muscle do not effectively respond to insulin stimulation. As a mechanism, hypertrophic adipose cells create hypoxic conditions, which leads to the death of adipocytes. Then, the dead adipocytes summon macrophages via released environmental factors. When macrophages have trouble clearing the debris of dead adipocytes, low-grade inflammation occurs. Macrophages and other immune cells release cytokines such as TNF-α, thereby targeting the insulin receptor substrate protein in serine residues and impairing tyrosine phosphorylation, leading to IR [40]. Moreover, dysbiosis complicates the inflammatory response in obese adipose tissue via 4-IBBL, which promotes IR through 4BL cells [41].

Among the various features of T2DM and metabolic disorders, IR is believed to be a prominent factor. Insulin plays a crucial role in energy balance and metabolism throughout the body, including the brain. When IR occurs, the normal functions performed by insulin, such as its neurotrophic function, become impaired, eventually leading to the death of neurons [42]. The critical role of insulin in neuron survival suggests that IR may be closely related to the development of neurodegenerative diseases such as AD. Furthermore, obesity, which is associated with reduced insulin sensitivity, can lead to peripheral hyperglycemia, contributing to cognitive dysfunction and neuron death in animal models [43].

Within the pathogenesis of T2DM, there are two kinds of IR: peripheral IR and brain IR. The former refers to IR in tissues outside the brain, as mentioned earlier, while the latter specifically refers to IR in certain brain regions, for instance, the hippocampus, cerebral cortex, cerebellum, and choroidal plexus [44]. Regarding brain IR, Zhao et al. discovered that apolipoprotein E4 (apoE4) impairs insulin signaling in the neurons of apoE-targeted replacement mice. Mechanically, apoE4 can trap insulin receptors inside the endosomes of neurons. Consequently, neurons become insensitive to insulin stimulation. It is also worth noting that the apolipoprotein E ε4 gene allele is the greatest genetic risk factor of late-onset AD [45]. Another in vivo study on two mouse models suggests that early AD is related to brain IR through different signaling pathways, such as the AKT signaling and ERK MAPK pathways. Consequently, brain IR further contributes to peripheral IR, establishing a detrimental feedback loop [46].

#### 2.1.2. Inflammation and Vascular Impairment

In the central nervous system, microglia cells play a pivotal role in AD local inflammation, secreting TNFα, IL-1β, IL-18, IL-6, chemokines, neurotransmitters, reactive oxygen species (ROS), and nitric oxide, which act as neuron destroyers [47].

Inflammation plays an important role in DM-related AD. AD and T2DM are both maladies characterized by systemic inflammation, and they encompass local inflammation. The overlap between systemic inflammation and local inflammation is represented by shared inflammatory markers, such as C-reactive protein, TNF-α, and interleukin-6 [48]. A clinical study tested the levels of chemokines such as IL-6, TNF-alpha, and IL-1-beta in AD and DM patients, and the serum level of these chemokines increased. Along with other results, this also suggests that low-grade systemic inflammation links AD and DM [49]. Moreover, a review summarized that the activation of the NLRP3 inflammasome represents a shared mechanism in AD and T2DM [50]. It is worth noting that elevated levels of inflammatory markers, ROS, and AGEs induced by T2DM can contribute to the disruption of the BBB [51].

The main battlefield of AD pathogenesis is in the brain. The BBB serves as a fortress for brain cells, and once it becomes damaged, chemicals such as inflammatory cytokines leak in, resulting in the activation of microglia cells. In neurodegenerative diseases, a subpopulation of microglia is called disease-associated microglia, which can be activated through TREM2 [52]. Within this subpopulation, there are two phenotypes of microglia cells, M1 (proinflammatory) and M2 (anti-inflammatory). Both phenotypes participate in different stages of AD [53]. In AD local inflammation, activated microglia cells play a pivotal role, secreting TNFα, IL-1β, IL-18, IL-6, chemokines, neurotransmitters, ROS, and nitric oxide, which act as neuron killers [47]. Moreover, microglia cells phagocytose apoptotic neurons, which is considered a protective mechanism. However, when excessive phagocytosis occurs, healthy synapses are “trimmed”, contributing to neurodegeneration [54].

#### 2.1.3. Glucose Metabolism Dysfunction and Mitochondrial Oxidative Stress

Glucose metabolism is a crucial process in which cells generate ATP and NADH using glucose as an energy source. Hence, this process is critical for the survival of cells, including neurons. A metabolomics study using nuclear magnetic resonance revealed a global disorder in glucose metabolism in APP/PS1 double-transgenic mice [55]. Additionally, in the early stages of AD pathogenesis, the most common type of disorder is amnestic mild cognitive impairment, which is characterized by a decrease in glucose metabolism, as observed in a longitudinal study [56]. Therefore, targeting disrupted glucose metabolism may be a promising approach to early-stage interventions for AD. Neurons cannot synthesize or store glucose. When glucose is required, plasma glucose travels to neurons through neurovascular coupling to neurons via glucose transporters. However, in neurodegenerative diseases such as AD, there is a noticeable decline in the neuron glucose intake because of the lowered glucose transporter 1 and 3 expression in the hippocampus and cortex [57,58]. Once glucose enters neurons, it undergoes aerobic glycolysis and enters the Krebs cycle, producing NADH and ATP. Eventually, the produced NADH interacts with the membrane of mitochondria, and then generates more ATP. This is known as oxidative phosphorylation [59]. Glycolytic enzymes play a vital role in these processes, and evidence suggests that the activity of these enzymes is reduced in AD patients [60]. When the activity of glycolytic enzymes declines, NADH and ATP production is reduced; consequently, a vicious cycle of mitochondrial dysfunction, oxidative stress, and calcium deregulation takes place [61].

Mitochondria are the major energy-metabolizing organelles in a cell and are also crucial for programmed cell death. In other words, a reduction in the number of mitochondria or in the activity of mitochondrial enzymes can lead to decreased energy metabolism. Interestingly, two critical metabolic pathways in neurons, oxidative phosphorylation and the Krebs cycle, are inhibited in the mitochondria in AD [62]. In addition to hypometabolism, mitochondrial dysfunction is also a pathogenesis process in AD brains. In healthy brains, mitochondrial dysfunction and metabolic deficiencies can be compensated by lysosome pathways, including the mitophagy of damaged mitochondria [63]. In AD brains, lysosomal deficiency is detected, which might exacerbate the hypometabolic state triggered by mitochondrial dysfunction [64]. As a result, hypometabolism and mitochondrial dysfunction cause oxidative stress and alter Ca^2+^ homeostasis, eventually leading to programmed neuron death [65]. Conversely, oxidative stress may also impair the function of mitochondria, thus forming another vicious cycle [66].

#### 2.1.4. Hyperglycemia and Advanced Glycation End Products

A population-based study showed that people diagnosed with severe hyperglycemia are liable to be living with AD [67]. Another study also found that poor glycemic control is associated with worse cognitive outcomes [68].

Hyperglycemia is common in untreated DM, which can be related to neuropathy. The hippocampus, an important part of the brain for working memory, and neuropathy in the hippocampus are found in AD. According to an in vivo study, after treating homozygous 3xTg-AD mice with 20% sucrose for 6 months, the hippocampal neurogenic reserve was reduced, and cognitive deficits were shown [69]. A longitudinal study included 266 individuals without DM or cognitive dysfunction. After controlling for confounding factors such as age and sex, the authors found that higher plasma glucose within the normal range is associated with hippocampal and amygdala atrophy based on an MRI scan [70]. This suggests that controlling raised blood sugar is a possible method of dealing with AD-related cognitive deficits. In addition to neuropathy in the hippocampus, neuropathy in the prefrontal lobe is another characteristic of AD. The prefrontal lobe is responsible for executive function. It has been reported that hyperglycemia is related to a higher HbA1c level in cognitively impaired patients, which suggests that hyperglycemia might reduce executive function in such patients [71]. At an early stage of AD, white matter hyperintensity is a sign of mild cognitive deficits. Based on a cross-sectional study, it is known that hyperglycemia is linked to brain atrophy and white matter hyperintensity, which also suggests that hyperglycemia is associated with cognitive decline and neuropathy [72].

Despite the current studies on neuropathy and cognitive decline in AD patients with hyperglycemia, the underlying mechanisms of this condition are still not fully understood. Several mechanisms have been discovered, including vascular impairment, microglial overactivation, and the involvement of receptors for advanced glycation end products (RAGE). Overactivated microglia are associated with neurodegeneration. A study reported that chronic hyperglycemia can induce microglia to exhibit a proinflammatory subtype known as M1, mediated by ERK5 signaling [73]. Moreover, high glucose impairs vesicle integrity in the brain, especially the BBB. Rao et al. found that the thrombin pathway contributes to damage in cultured microvascular endothelial cells of the human brain [74]. Furthermore, RAGE is the receptor of AGEs. AGEs are generated through a non-enzymic process between reduced sugar and aminos and are produced in larger quantities in hyperglycemia and DM patients, compared to those without these conditions. On the one hand, when AGEs are produced in the neurons it prompts oxidative stress and neuroinflammation; on the other hand, extracellular AGEs could affect neuron function after binding to RAGE [75]. Upon injecting AGEs into DM model mice, the mice showed AD-like features, such as decreased memory, suggesting that AGEs are contributing factors in the comorbidity of DM and AD [76].

Hyperglycemia is a detrimental element in AD, and hypoglycemia is not beneficial either. Recurrent moderate hypoglycemia is common in treated DM, and a study of streptozotocin-induced diabetic APP/PS1 mice indicated that recurrent hypoglycemia could promote AD via the damaged TRPC6/GLUT3 pathway [77]. Another study also discovered that non-severe hypoglycemia has an adverse effect on the cognition of T2DM patients [78]. Conversely, a different in vivo study indicated that only severe hyperglycemia shows damage to neurons and cognition, while recurrent moderate hypoglycemia does not show damage to cognition or hippocampal neurons. Ironically, recurrent moderate hypoglycemia prepares the brain to better handle possible neuron damage caused by severe hypoglycemia [79]. It is worth noting that the possible mechanisms underlying this process have not yet been elucidated.

### 2.2. Obesity

Obesity is associated with a variety of factors, including genetic composition, diet, and lack of exercise [80]. One of the main mechanisms via which obesity contributes to AD is IR. We have already provided details of IR in the section on T2DM. Herein, we will discuss other studied mechanisms that may underlie the obesity-to-AD pathogenesis process, for instance, oxidative stress, ER stress, inflammation, and leptin [81].

#### 2.2.1. Inflammation

Systemic inflammation plays a role in the pathogenesis of AD, and chronic low-grade systemic inflammation is the main feature of obesity. An experiment conducted on obese C57BL/6 mice fed on a high-fat diet confirmed that obesity in aging is associated with an increase in the systemic inflammatory status, which exacerbates the destruction of the BBB [82]. Research has demonstrated that adipose tissue can promote the secretion of some cytokines, such as tumor necrosis factor-α, interleukin-1β, interleukin-6, and chemokine (C-C motif) ligand 2, and thus recruit macrophages and lymphocytes [83]. Then, secreted tumor necrosis factor-α and interleukin-6 can reduce lipoprotein lipase activity, thereby increasing blood lipid levels [84]. It is worth noting that a high blood lipid level and a high-fat diet are associated with dysbiosis in the gut, and this can lead to impaired lipid metabolism [85]. Furthermore, these adipokines and cytokines are able to act together on distant organs, especially the brain, promoting the production of brain cytokines by activating endothelial and glial cells, particularly microglia [81].

Neuroinflammation is also linked to AD pathogenesis, and neuropathy in the hippocampus is one of the key processes of AD pathogenesis. According to an in vivo study, after consuming a high-fat diet for three days, molecular changes such as neuroinflammation, ER stress, and the apoptotic signal were found in the hippocampus of C57BL/6J mice [86]. Another study also suggested that a high-fat diet induces inflammation, represented by microgliosis and astrocytosis in the hippocampus of THY-Tau22 male mice [87]. Obesity is accompanied by an array of metabolic disorders, and the AMPK pathway is critical in metabolic homeostasis. Mechanically, the AMPK pathway regulates glucose and lipid metabolism. Upon activation, it ameliorates oxidative stress, IR, and mitochondrial dysfunction [88]. Liu et al. found that activated C/EBPβ/AEP signaling inhibits AMPK signaling, which is related to an AD-like pathology. After feeding Thy1-C/EBPβ Tg mice with a high-fat diet to induce DM and obesity, they found neuroinflammation characterized by gliosis and microglia activation, which activates C/EBPβ/AEP signaling. After deleting AEP, DM, obesity, and AD-like pathogenesis were lessened [89].

#### 2.2.2. Leptin

In addition to inflammation, the role of adipokines in linking obesity and AD has also been discovered. Adipose tissue releases some adipokines into the circulation, such as leptin. Leptin is transported into the brain through leptin receptors in the BBB or tanycytes [90]. Leptin receptors broadly reside in brain regions such as the hypothalamus and hippocampus. Moreover, leptin regulates food consumption and energy expenditure through neuroendocrine signaling in the hypothalamus, and at the same time, it can enhance the plasticity and strength of synapses and prevent tau phosphorylation [91].

According to a cross-sectional study, elevated serum levels of adipokines, including leptin, are observed in AD patients compared to patients with mild cognitive impairment (MCI) [92]. Another study suggests that the degree of dementia is negatively associated with serum leptin levels in AD patients [93]. However, a cross-sectional study by Teunissen et al. concluded that the serum level of leptin is not related with cognitive decline in AD or vascular dementia patients [94]. Although no changes in CSF leptin levels are observed in AD patients, alterations in leptin signaling have been documented. It has been suggested that leptin plays a role in early-stage rather than late-stage AD [95]. Concerning leptin signaling, another study suggested that the alternation of leptin signaling in the hippocampus is a characteristic process in AD pathogenesis [96]. In terms of the mechanism, an in vitro study found that leptin could ameliorate mitochondrial dysfunction induced by glucose–serum deprivation and Aβ_1–42_ stimulation [97]. Considering the above, there is still a heated discussion about the role of leptin in AD pathogenesis, and further research is needed to fully understand its implications.

#### 2.2.3. Endoplasmic Reticulum Stress

ER stress is implicated in the progression of AD neuropathy and is responsible for cognitive decline in AD. It is activated by a spectrum of diseases, for instance, diabetes mellitus and obesity [98]. The hippocampus contains progenitor cells in the dentate gyrus and the sub-granular zone, and altered neurogenesis in the hippocampus is a cornerstone of early-stage AD. An in vivo study suggests that long-term obesity can induce ER stress in the hippocampus DG of C57BL/6J and APP23 mice [99]. This suggests that ER stress might play a critical role in early-stage AD.

#### 2.2.4. Mitochondrial Oxidative Stress and Impaired Blood–Brain Barrier

Mitochondrial oxidative stress in brain microvascular endothelial cells represents another link between obesity and AD. Roh et al. found that mitochondrial oxidative stress can impair the integrity of the BBB [100]. However, the underlying mechanisms have still not been elucidated. Considering the roles of insulin and the mitochondria in energy metabolism, mitochondrial dysfunction can be regulated through insulin-mediated PI3K signaling. Hence, epithelial mitochondrial dysfunction might be induced by epithelial IR. Nagano et al. knocked out insulin receptors in brain microvascular endothelial cells, and they found that insulin signaling is critical in BBB function and maintenance [101]. After the disruption of mitochondria by ROS, the debris is recognized by pattern recognition receptors such as NLRP3. Eventually, the NLRP3 inflammasome is activated, leading to pyroptosis. In summary, under the influence of mitochondrial oxidative stress, the BBB becomes more permeable. After the permeability of the BBB increases, cytokines produced by adipose tissue find their way across it. With so many cytokines crossing the BBB, it can affect the CNS, including a loss of synapses, hypothalamic dysfunction, and impaired cognition and neurodegeneration [102]. Notably, a clinical study found a strong correlation between alterations in BBB permeability in the human hippocampus and cognitive dysfunction [103].

#### 2.2.5. Gut Microbiota

It is well known that the gut and the brain are associated with each other through many bioactive compounds secreted by both, hence proposing the name “gut–brain axis” or “brain–gut axis”. Bioactive compounds secreted by the gut microbiota incorporate neurotransmitters and neuromodulators such as glutamate, short-chain fatty acids, and serotonin. The connections between them are diverse, and include pathways such as neural, metabolic, and endocrine pathways [104]. Therefore, it is often believed that neurodegenerative diseases such as AD are associated with the gut, and there is indeed some etiological evidence suggesting that AD is associated with a disturbance in the gut microbiota, called dysbiosis [105]. Moreover, dysbiosis is also a pivotal starter in many other diseases such as T2DM [106] and obesity [107].

In addition to the neuroinflammation hypothesis of AD pathogenesis, where microglia are overactivated, a recent study suggests that amino acids such as phenylalanine and isoleucine in dysbiosis are key to the infiltration of peripheral immune cells such as B cells, dendritic cells, and natural killer cells in the brain. Moreover, dysbiosis has also been found to evoke microglia to a destructive state called the M1 subtype through peripheral Th1 cells, and it is a critical contributor to Th1/M1 predominant inflammation in AD [108]. Another study suggests that the gut microbiota in AD patients can activate the NLRP3 inflammasome in intestinal cells; then, the intestinal cells secret an array of cytokines that can pass through the BBB, which causes central inflammation [109]. On the contrary, short-chain fatty acids secreted by the gut microbiota, such as acetate, propionate, butyrate, formate, and valerate, not only show anti-inflammatory activity in the peripheral system, but also inhibit the production of inflammatory cytokines and cytotoxins by microglia-like cells [110]. Moreover, another study suggests that sodium butyrate relieves neuroinflammation by inhibiting microglia and ameliorates synaptic plasticity in 5XFAD mice [111].

In oxidative stress and neuroinflammation, a molecule called Nox2 plays a pivotal role. A study found that LPS released by the gut microbiota can activate Nox2; thus, LPS are associated with oxidative stress and neuroinflammation in AD through Nox2 activation [112]. Another study suggests that the oral intake of lactobacilli and bifidobacteria improves spatial performance and balances oxidant/antioxidant biomarkers in the β-amyloid-administered animals [113]. Furthermore, other authors suggest that sodium butyrate suppresses Nox2 and upregulates SOD1 via the p21/Nrf2 pathway [114].

Regarding lipid metabolism, the deregulation of lipid homeostasis considerably contributes to the onset and development of AD. Bonfili et al. found that gut microbiota adjustment through the combination of probiotic strains can ameliorate neuroinflammation and oxidative stress by regulating the plasma lipid profile [115]. Moreover, glucose metabolism is also a contributor in AD onset and progression. A study suggested that lactic acid bacteria and bifidobacterial supplementation increases GLUTs in 3×Tg-AD mice and downregulates phosphorylated AMPK and Akt, thus suggesting that gut microbiota manipulation ameliorates impaired glucose metabolism [116]. Considering that dysbiosis is closely related to metabolic syndromes such as DM and obesity, a gut metabolite called trimethylamine N-oxide has been discovered. It was used to induce oxidative stress and cause cognitive impairment in 3×Tg-AD mice [117].

In summary, manipulating dysbiosis can have a positive effect on the onset and progression of AD by regulating oxidative stress, inflammation, and metabolism. It also offers a potential target for AD intervention.

## 3. Biomarkers

AD is so detrimental that the early identification of markers that can anticipate the onset and progression of AD is critical. On the one hand, DM is an independent risk factor for AD, and early AD may manifest with DM-like symptoms. Therefore, it is worth searching for biomarkers that can predict changes in the onset and progression of AD associated with DM. These biomarkers can be detected in different body fluids. The first category is biomarkers present in cerebrospinal fluid, such as pTau, Aβ_42_, sLRP1, and autotaxin, and their elevation is related to the occurrence and development of AD [118,119]. Another class of biomarkers, such as GGT, can be detected in serum. Furthermore, gamma-glutamyl transferase (GGT) plays a role in oxidative stress and inflammation, and a nation-wide study found that GTT increases the incidence of dementia in DM patients independently of other factors [120]. Additionally, plasma biomarkers such as clusterin can be measured. One study demonstrated that clusterin levels were elevated in both AD and DM patients, and its elevation is correlated with disease severity [121]. In addition to molecular biomarkers, a new technology, called microperimetry, can identify DM individuals who are more likely to develop AD by assessing their retinal sensitivity [122].

Mild cognitive impairment (MCI) is often regarded as an early stage of AD, and it is also considered as an independent risk factor for AD. Individuals with MCI have a higher likelihood of developing AD after several decades. A study found that, compared with Aβ_1-42_/Aβ_1-40_, rGSK-3 is the most effective biomarker in identifying MCI from T2DM patients [123]. Another study suggests that a lower level of an adipocytokine called adiponectin is related to development from MCI to AD. Neurofilament light chain is a cytoskeletal protein in neural axons that becomes elevated when neurodegeneration occurs. A study found that elevated plasma levels of NfL may serve as a potential biomarker for MCI in individuals with DM [124]. Moreover, another study suggested that plasma neuroexosomal NADH ubiquinone oxidoreductase core subunit S3 and succinate dehydrogenase complex subunit B are elevated in the early stage of AD [125]. One more adipokine, called resistin, is also a biomarker of T2DM-related cognitive decline [126]. Furthermore, Wang et al. found that a high plasma PAI-1 level and a low tPA/PAI-1 molar ratio are correlated with MCI in DM patients [127]. A muti-center study suggested that aging, the expression of ApoE ε4, the activation of GSK-3beta in the peripheral circulation, and an increased olfactory score are indicative biomarkers of MCI in DM patients, and they can be used together to achieve a higher diagnostic accuracy [128]. In addition to molecular biomarkers, a new technology, called microperimetry, can identify DM individuals who are more likely to develop AD by assessing retinal sensitivity [122]. A summary of related biomarkers are shown in Table 1.

## 4. Properties of 10-Hydroxy-2-decenoic Acid

### 4.1. Anti-Neurodegeneration and Immunomodulation

The process of pyroptosis, a form of cell death associated with inflammation, has been implicated in the development of AD and MetS. The NLRP3 inflammasome is responsible for initiating pyroptosis. A study has shown that 10-HDA can enhance the function of the colonic barrier by inhibiting the NLRP3 inflammasome-mediated apoptotic pathway [138]. Additionally, You et al. discovered that 10-HDA can alleviate neuroinflammation in microglia BV2 cells through the FOXO1-mediated autophagy pathway, indicating that it may be a promising agent for various neuroinflammation-associated diseases [139]. Damage to the BBB is considered a crucial factor in the pathogenesis of AD. You et al. demonstrated that 10-HDA can inhibit the degradation of tight-junction proteins, reduce BBB permeability, and protect the integrity of the BBB through the AMPK/PI3K/AKT pathway [140].

Furthermore, recent research has suggested that 10-HDA possesses immunomodulatory effects as it binds to pattern recognition receptors. One of these receptors is TLR4, which serves as a sensor for damage-associated molecular patterns [141]. Farshid et al. found that 10-HDA acts as an antagonist to inhibit immune cell activation induced by TLR4 [142]. These findings underscore the immunomodulatory properties of 10-HDA and its potential role in regulating immune responses.

### 4.2. Antitumor

Zafer et al. found that 10-HDA elevates the expression of caspase 3, Bax, and miR-34a. Then, it increases necrotic and apoptotic human hepatoma cells [143]. Albalawi et al. found that 10-HDA, in combination with cyclophosphamide, showed antitumor effects against Ehrlich solid tumors [144]. Lin et al. suggested that 10-HDA induces cell cycle arrest and apoptosis in A549 human lung cancer cells through the MAPK, STAT3, NF-κB, and TGF-β1 signaling pathways [145]. These studies highlight the potential antitumor properties of 10-HDA and its ability to induce cell death and inhibit cancer cell growth. Properties of 10-HDA are shown in Table 2.

### 4.3. Metabolic Adjusting Properties

**Figure 2:**
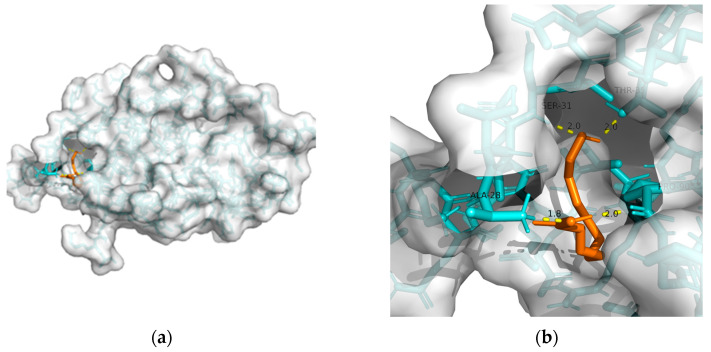
Docking results between 10-HDA and GLP-1R: (**a**) A gross view of the optimal conformation between GLP-1R and 10-HDA after molecular docking. (**b**) Details of the binding site be-tween GLP-1R and 10-HDA. Here, 10-HDA is represented as orange sticks, while GLP-1R is represented as a grey surface. And amino acid residues are represented as blue sticks, while hydrogen bonds are represented as dashed yellow sticks. Four hydrogen bonds are shown in the image, and 10-HDA is rooted in the binding pocket of GLP-1R.

**Figure 3:**
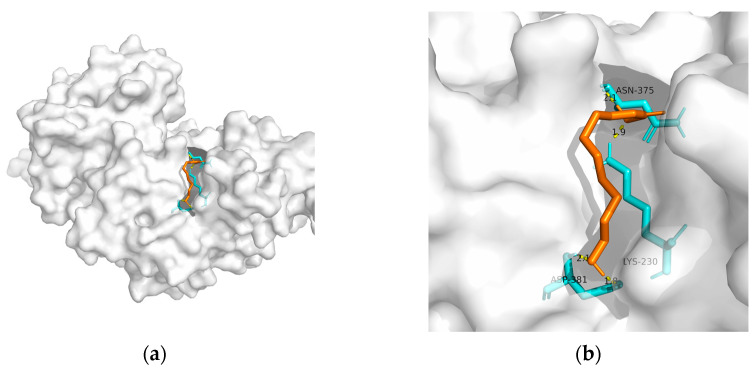
Docking results between 10-HDA and PPAR-γ: (**a**) A gross view of the optimal conformation between PPAR-γ and 10-HDA after molecular docking. (**b**) Details of the binding site be-tween PPAR-γ and 10-HDA. Here, 10-HDA is represented as orange sticks, while PPAR-γ is represented as a grey surface. And amino acid residues are represented as blue sticks, while hydrogen bonds are represented as dashed yellow sticks. Four hydrogen bonds are shown in the image, and 10-HDA is rooted in the binding pocket of PPAR-γ.

**Figure 4:**
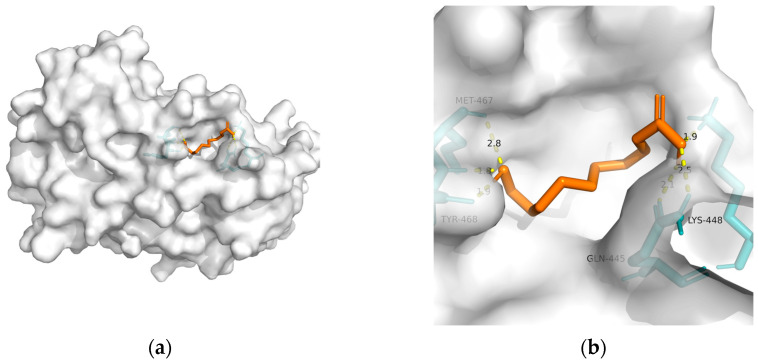
Docking results between 10-HDA and PPAR-α: (**a**) A gross view of the optimal conformation between PPAR-α and 10-HDA after molecular docking. (**b**) Details of the binding site be-tween PPAR-α and 10-HDA. Here, 10-HDA is represented as orange sticks, while PPAR-α is represented as a grey surface. And amino acid residues are represented as blue sticks, while hydrogen bonds are represented as dashed yellow sticks. Six hydrogen bonds are shown in the image, and 10-HDA is rooted in the binding pocket of PPAR-α.

**Figure 5:**
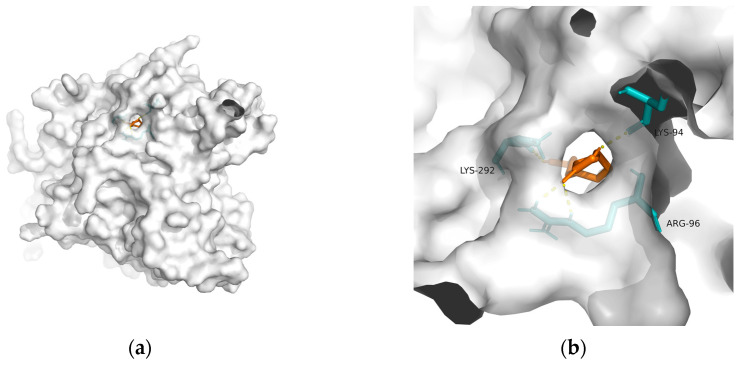
Docking results between 10-HDA and GSK-3: (**a**) A gross view of the optimal conformation between GSK-3 and 10-HDA after molecular docking. (**b**) Details of the binding site between GSK-3 and 10-HDA. Here, 10-HDA is represented as orange sticks, while GSK-3 is represented as a grey surface. And amino acid residues are represented as blue sticks, while hydrogen bonds are represented as dashed yellow sticks. Four hydrogen bonds are shown in the image, and 10-HDA is rooted in the binding pocket of GSK-3.

**Figure 6:**
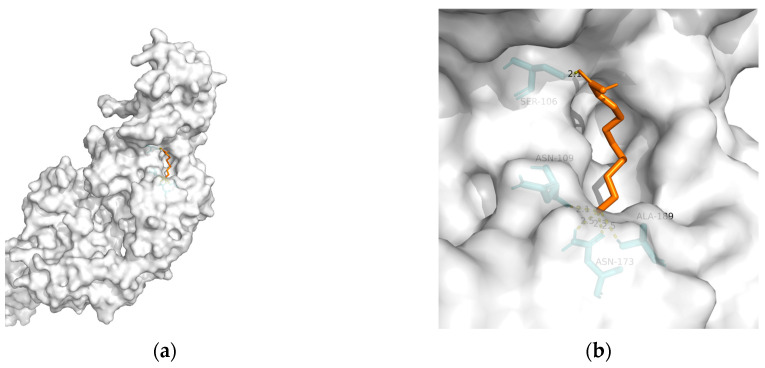
Docking results between 10-HDA and TREM2: (**a**) A gross view of the optimal conformation between TREM2 and 10-HDA after molecular docking. (**b**) Details of the binding site be-tween TREM2 and 10-HDA. Here, 10-HDA is represented as orange sticks, while TREM2 is represented as a grey surface. And amino acid residues are represented as blue sticks, while hydrogen bonds are represented as dashed yellow sticks. Five hydrogen bonds are shown in the image, and 10-HDA is rooted in the binding pocket of TREM2.

As mentioned previously, IR plays a critical role in MetS-related AD. Hu et al. found that 10-HDA increases insulin sensitivity by upregulating the PI3K/Akt pathway in the liver [146]. In addition to adjusting glucose metabolism, Fan et al. found that 10-HDA may protect •OH-damaged vascular smooth-muscle cells by adjusting energy metabolism and protein metabolism [148]. Considering the above, T2DM and obesity are linked to AD through a spectrum of mechanisms, whereas no metabolic adjusting targets have been studied. Herein, we screened several macromolecules that might be targets for MetS-related AD intervention. Then, we performed molecular docking between these macromolecules and 10-HDA. The results are shown in Table 3 and Figure 2, Figure 3, Figure 4, Figure 5 and Figure 6.

GLP-1 is an insulinotropic incretin hormone that plays a role in inhibiting beta cell death. It is also important to understand that GLP-1 mimetics can cross the BBB. Overall, this suggests that the GLP-1 pathway overlaps with the insulin pathway, and it could compensate for the DM, obesity, or AD damage to the insulin pathway. A review also suggests that GLP-1 serves as an ameliorator of ER stress, IR, CNS inflammation, mitochondrial dysfunction, etc., which sheds light on AD intervention [160]. A GLP-1R agonist, liraglutide, has been approved for obesity treatment [161]. Furthermore, GLP-1R agonists are currently used to treat T2DM [162].

PPARs are a family of type II nuclear receptors and transcription factors, which include PPAR-α, PPAR-δ, and PPAR-γ. They have been found to be involved in metabolic syndromes such as DM and obesity [163]. PPAR-γ is considered a general sensor for nutrients and lipids, residing in the nucleus, and playing a role in mediating responses to nutrients and hormones. It is also implicated in metabolic syndrome [164]. Lin et al. found that Aβ-induced ER stress can be alleviated through PPAR-γ signaling [165]. PPAR-α is another nuclear receptor belonging to PPARs. Many agonists, such as Gemfibrozil, are synthesized or found to tackle AD in clinical trials [166]. In the hippocampus, according to Avik Roy et al., PPAR-α resides in CA1, CA2, CA3, and the dentate gyrus in the brains of mice and monkeys, and the absence of PPAR-α in both wild-type mice and hippocampal neurons leads to an insufficient calcium influx and reduced hippocampal plasticity-related molecules, such as GluR1 and NR2A [167]. Moreover, PPAR-α participates in energy metabolism and fatty acid regulation in mitochondria. Furthermore, PPAR-α also plays a role in oxidative stress [168].

There are two isoenzymes in the GSK-3 family, called GSK-alpha and GSK-beta. GSK-beta, most importantly, acts as a blood glucose regulator in DM. It is also one of the vital factors that leads to IR and insulin deficiency [169]. Moreover, it is reported that GSK-3 plays an important role in energy metabolism and apoptosis. It is known as a negative regulator of inflammation in microglial cells, macrophages, and dendrite cells, and is also involved in osteoclast development. Furthermore, Min Park et al. suggested that TREM2 promotes IR and facilitates diet-induced obesity, which makes TREM2 a possible target in treating obesity and DM [170]. Zhang et al. used a DM rat model treated with a combination of HFD and a low dose of STZ. They found that TREM2 negatively regulates the p38 MAPK-mediated inflammation response and neuronal cell death in the hippocampus and cortex in cognitively impaired DM rats [171].

## 5. Summary and Outlook

MetS and AD have a significant global impact. They are connected through both epidemiological and pathological aspects. In the connection between MetS and AD, low-grade systemic inflammation is at the core, IR represents a bridge, and BBB damage represents a hallmark on the bridge. In other words, systemic IR is induced by low-grade systemic inflammation, especially inflammation in adipose tissue. Subsequently, IR leads to hyperglycemia and dysfunction in energy metabolism. Moreover, IR causes dysbiosis in the gut as well as more AGEs and ROS in the circulation. Inflammatory cytokines, AGEs, and ROS compromise the BBB, and then they infiltrate the brain matter. Afterwards, neurons are scarred or killed by inflammatory cytokines, AGEs, ROS, or microglia cells. However, considering that the underlying etiology of AD has not yet been pinpointed, it is important to introduce a possible intervention nutrient to relieve these diseases at an early stage [5].

In RJ, 10-HDA is the most abundant fatty acid, which shares similar properties with RJ. Moreover, compared with RJ, 10-HDA has little allergy risk. Regarding its metabolic adjusting property, Takikawa et al. found that 10-HDA promotes glucose uptake by translocating glucose transporter 4 to the plasma membrane in mice skeletal muscle [172]. However, the metabolic adjusting capability of 10-HDA remains vague. Herein, we screened a few macromolecules (GLP-1R, PPAR-gamma, PPAR-alpha, GSK-3, and TREM2) that might be targets for MetS-related AD intervention. Our results suggest solid binding between 10-HDA and GLP-1R, PPAR-γ, PPAR-α, GSK-3, and TREM2. Moreover, GLP-1R might be the most promising intervention target for 10-had, with the lowest free binding energy being −24.27 KJ/mol.

Interventional methods such as nutrimental supplementation are ideal ways of dealing with chronic disease related to age based on common knowledge, and 10-HDA is easily accessible from RJ. Our research suggests that 10-HDA might represent an intervention nutrient that can deal with AD, DM, and obesity by targeting GLP-1R. However, it is important to note that the study of 10-HDA is currently limited, and further in vitro and in vivo studies are necessary to fully understand its potential benefits.

## Figures and Tables

**Table 1:** Biomarkers of MetS-related AD.

Biomarker	Fluid Source	Function	Results	References
pTau	Cerebrospinal fluid	Correlated with the intensity of neurodegeneration and neurofibrillary-tangle pathology, respectively	The level of total tau was raised in APOE E4+ AD patients with DM	[31,129]
Aβ_42_	Cerebrospinal fluid	Forms neurotic plaques and causes impaired synaptic plasticity and neuronal cell death	CSF levels of Aβ_42_ were higher in patients with type 1 diabetes than in controls	[118,130]
sLRP1	Cerebrospinal fluid	A member of the LDL receptor family	CSF levels of LRP1 were higher in patients with type 1 diabetes than in controls	[118,131]
autotaxin	Cerebrospinal fluid	Hydrolyzes lysophosphatidylcholine into lysophosphatidic acid	Autotaxin levels were significantly higher in MCI and AD	[119,132]
GGT	Serum	Cellular antioxidant, glutathione metabolism	Higher levels of GGT activity were correlated with dementia in patients with DM	[133]
GSK-3beta	Serum	A serine/threonine kinase	Serum level of GSK-3β protein was higher in the T2DM-MCI group than the T2DM-nMCI group	[128]
Clusterin	Plasma	Participates in several kinds of cellular processes, such as synaptic regulation, lipid transport, extracellular misfolded protein clearance, and complement inhibition	Clusterin increased with disease severity in AD and DM patients	[121,134]
NfL	Plasma	An intermediate filament of the neuronal cytoskeleton	Elevated blood levels of NfL can be used to screen for AD	[135]
NDUFS3	Plasma	Subunits of electron transport chain complex	NDUFS3 was lower in patients with T2DM with AD dementia and progressive MCI	[125]
SDHB	Plasma	Subunits of electron transport chain complex	SDHB was lower in patients with T2DM with AD dementia and progressive MCI	[125]
resistin	Plasma	Play a role in energy homeostasis and regulation of metabolism	Higher plasma levels of resistin were associated with a decreased risk of dementia and AD	[136]
PAI-1	Plasma	A serine protease inhibitor and cell senescence marker	Plasma PAI-1 protein levels were increased in the elderly and in the AD brain	[137]

**Table 2:** Mechanisms related to 10-HDA properties.

Related Mechanisms	Results	Model	References
Apoptosis	Inhibits apoptosis in human hepatoma cells.	Human hepatoma cell line.	[143]
Inflammation Antioxidation	Hypoglycemic effects on diabetic mice, through the PI3K/AKT/GSK3β signaling pathway.	Diabetic C57BL/6J mice.	[146]
Inflammation	Blocks TLR4.	HEK293T cells with high TLR4 expression.	[142]
Inflammation Antioxidation	Increases serum concentrations of immunoglobulin G at d 21, as well as IgM and interleukin-10 at d 42, while decreasing the levels of tumor necrosis factor-α.	Broiler Chickens.	[147]
Inflammation Antioxidation	Inhibits inflammasome-mediated pyroptosis induced by LPS/ATP.	Male C57BL/6 mice.	[138]
Antioxidation Energy metabolism Vascular function	Maintains vascular health via scavenging •OH.	Vascular smooth-muscle cells.	[148]
Inflammation	Attenuates the secretion of TNF-α, IL-6, and IL-1β.	Macrophages (RAW264.7 cells)	[149]
Antimicrobial	Decreases biofilm viability and effectively eradicates mature biofilms.	*Staphylococcus aureus*.	[150]
Antitumor	Decreases tumor volume, tumor markers (AFP and CEA), and TNF-α level.	Female Swiss albino mice.	[144]
Immunomodulation	Blocks TLR4.	Dendritic cells	[141]
Antimicrobial Antioxidation	Shows antioxidant and antimicrobial activity.	*Statens Seruminstitut* Rabbit Cornea cell culture line.	[151]
Apoptosis Antioxidation	Induces apoptosis through ROS-mediated MAPK, STAT3, NF-κB, and TGF-β1 signaling pathways.	A549 human lung cancer cells.	[145]
Autophagy	Protects against neuroinflammation through FOXO1-mediated activation of autophagy.	Microglial BV-2 cells (LPS-induced).	[139]
Immunomodulation	Improves immunity in the thymus and spleen	BALB/c mice.	[152]
Vascular function	Improves blood–brain barrier dysfunction by activating the AMPK/PI3K/AKT pathway.	C57BL/6 mice (LPS-stimulated).	[140]
Insulin signaling Anti-adipogenesis	Inhibits cAMP/PKA pathway and p-Akt- and MAPK-dependent insulin signaling pathway.	3 T3-L1 adipocyte cell line.	[153]
Inflammation Antimicrobial	Modulates interleukin-8, IL-1β, and tumor necrosis factor-alpha.	WiDr cell.	[154]
Melanogenesis inhibitor	Inhibits the activity of tyrosinase and the expression of tyrosinase-related protein 1, TRP-2, and microphthalmia-associated transcription factor.	B16F1 melanoma cells.	[155]
Antioxidation	Decreases tumorigenic potential of various tumor cells.	Human colorectal adenocarcinoma cells.	[156]
Insulin-like signaling	Extends lifespan through dietary restriction signaling.	*Caenorhabditis elegans*.	[157]
Antioxidation	Reduces the UVA-induced activation of the JNK and p38 MAPK pathways.	Human dermal fibroblasts.	[158]
Inflammation	Increases procollagen type I and TGF-β1 production.	Human dermal fibroblasts.	[159]

**Table 3:** The results of molecular docking (optimal conformation).

Macromolecule	PDB	DeltaG (KJ/mol)	RMSD (Å)	Binding Site (Number)	Hydrogen Bonds
GLP-1R	3c5t	−24.27	2.193	Ala^28^, Ser^31^, Thr^35^, and Pro^90^	4
PPAR-gamma	2q59	−20.59	0.956	Asn^375^, Lys^230^, and Asp^381^(2)	4
PPAR-alpha	3vi8	−22.47	1.598	Tyr^468^(2), Met^467^, Gln^445^(2), and Lys^448^	6
GSK-3	1q5k	−23.81	1.556	Lys^292^, Lys^94^, and Arg^96^	4
TREM2	6yye	−12.38	1.212	Ser^106^, Asn^109^, Asn^173^(2), and Ala^189^	5

## Abbreviations

AD	Alzheimer’s disease
MetS	Metabolic syndromes
DM	Diabetes mellitus
T1DM	Type 1 diabetes mellitus
T2DM	Type 2 diabetes mellitus
10-HDA	10-Hydroxy-2-decenoic acid
RJ	Royal Jelly
ER	Endoplasmic reticulum
IR	Insulin resistance
MCI	Mild cognitive impairment
GLP-1R	Glucagon-like peptide-1 receptor
PPARs	Peroxisome proliferator-activated receptors
GSK-3	Glycogen synthase kinase-3
TREM2	Triggering receptor expressed on myeloid cells 2
BMI	Body mass index
BBB	Blood–brain barrier
AGEs	Advanced glycation end products
RMSD	Root mean square deviation
